# Vitamin D binding protein gene polymorphisms in Ugandan tuberculosis patients and household contacts: A pilot study

**DOI:** 10.12688/f1000research.160839.1

**Published:** 2025-02-04

**Authors:** Acen L. Ester, Joloba L. Moses, Ashraf Akintola, Rizwana Begum Syed Nabi, Irene Andia Biraro, William Worodria, Alfred Okeng, Kelvin Bwambale, Mudarshiru Bbuye, David Patrick Kateete

**Affiliations:** 1 Department of Physiology, School of Biomedical Sciences, College of Health Sciences, Makerere University, Kampala, POBOX 7072, Uganda; 2 Department of Immunology and Molecular Biology, School of Biomedical Sciences, College of Health Sciences, Makerere University, Kampala, POBOX 7072, Uganda; 3 Department of Biomedical Convergence Science and Technology, School of Industrial Technology Advances, Kyungpook National University, Daegu, 41566, South Korea; 4 Department of Southern Area Crop Science, National Institute of Crop Science, Rural Development Administration, Miryang, 50424, South Korea; 5 Department of Internal Medicine, School of Medicine, College of Health Sciences Unit, Makerere University, Kampala, POBOX 7072, Uganda; 6 Department of Internal Medicine, Pulmonary Division, Mulago National Referral Hospital, Kampala, Uganda; 7 Department of Molecular Biology and Biotechnology, School of Bio-security and Laboratory Sciences, College of Veterinary Medicine, Makerere University, Kampala, POBOX 7072, Uganda; 8 Department of Biosecurity Ecosystems and Veterinary Public Health , School of Bio-security and Laboratory Sciences, College of Veterinary Medicine, Makerere University, Kampala, POBOX 7072, Uganda; 9 Makerere Lung Institute, College of Health Sciences, Makerere University College of Health Sciences, Kampala, POBOX 7072, Uganda

**Keywords:** Vitamin D, binding protein, gene, polymorphisms, Tuberculosis

## Abstract

**Background:**

Tuberculosis remains a significant global public health concern. Genetic variants influence the distribution of vitamin D in circulation, leading to vitamin D deficiency. The two extensively studied non-synonymous D-binding protein nucleotide polymorphisms rs7041 and rs4588 were found in different populations. This study aimedto compare the frequency distribution of vitamin D binding protein gene polymorphisms in patients with active Ugandan tuberculosis, individuals with latent tuberculosis infection, and those with no tuberculosis infection.

**Methods:**

This pilot studyselected 102 samples, including 52 active tuberculosis patients, 23 latent tuberculosis individuals, and 27 individuals without tuberculosis infection, from a previous cross-sectional study. Vitamin D binding protein genesrs7041 and rs4588 were genotyped usingPolymerase Chain reaction and Sanger sequencing. vitamin D binding protein gene polymorphisms were identified using BioEditsoftware. 7.2 ( http://www.mbio.ncsu.edu/BioEdit/bioedit.html)

**Results:**

This study revealed no significant differences in DBP genetic polymorphisms among the study groups. The frequency distribution of the DBP gene has been reported to be 97% Gc1F, 2% Gc2, and 1% Gc1S. The frequency distribution among patients with TB was 96.2% for Gc1F, 0% for Gc1F, and 3.8% for Gc2. Among the LTBI cases, 95.7% were Gc1F, 4.3% were Gc1S, and 0%were Gc2. The Hardy-Weinberg equilibrium analysis was in equilibrium, D’= 0. P=0.2

**Conclusions:**

The Gc1F genotype was predominantly found in the study population, with no difference in the frequency distribution according to TB status. However, minor alleles appear to be associated withan increased risk of active and latent TB. Therefore, we recommend larger studies in different populations to elucidate the functional significance of these minor alleles and their roles in TB pathogenesis.

## Introduction

Vitamin D Binding Protein (DBP), also known as group-specific component (Gc), is one of the most prevalent and significant carrier proteins of vitamin D metabolites, accounting for an estimated 85-90% of the total metabolite. ^1– 4^ The unbound fraction, which is the free fraction, was estimated to be less than 1%, whereas the albumin-bound fraction was approximately 10-15%. ^5^ DBP, amember of the albumin family, is synthesized in the liver. ^6^ This protein is considered responsible for vitamin D deficiency in target cells, as the bound fraction has a minimal impact on target cells. ^6, 7^ Other functions of DBP include actin scavenging, macrophage activation, and fatty acid transport. ^1^

The highly polymorphic DBP gene is located at 4q12-q13, with over 120 variants. ^8^ These genetic variations,affect the circulatory distribution of vitamin D, which leads to vitamin D deficiency. ^3^ In various populations of the world, the two extensively studied non-synonymous DBP single nucleotide polymorphisms (SNPs) rs7041 and rs4588 exhibit variable distributions. ^2^ These variations are located in exon 11, where 7041 encodes c.1296 T>G p.Asp416Glu, while rs4588 encodes c.1307 C>A p.Thr420Lys. ^9^ These two variations give rise to three polymorphic isoforms, which are known to differ by lineage and include Gc1F, Gc1S and Gc2. ^10, 11^ The wild type of these SNPs is Gc1Fgenotype variations in the in Gc1F, D416E, and T420K result in, the Gc1S and Gc2 genotypes, respectively. ^12^ Gc2 is found at locus rs4588 while Gc1F and Gc1S are found at locus rs7041. ^8^ Previous studies have shown that people who have the rs7041 G allele as a substitute for the T allele and the rs4588 A allele instead of the C allele have higher levels of DBP and a higher affinity for vitamin D, consequently resulting in lower free and bioavailable vitamin D levels. Consequently, the DBP role controlling total, free, and bioavailable vitamin D is crucial in immunity and influences progression of disease. ^13^

Studies have documented that vitamin D deficiency contributes to TB susceptibility, and individuals with deficiency are at a high risk of developing TB. ^14^ Therefore, vitamin D status is implicated in the response to M. tuberculosis, and is genotype-dependent, varying across geographical areas. ^15^

The wild-type Gc1F genotype is predominantly found in the African population, with a low frequency of Gc2 and Gc1S and is associated with low levels of vitamin D. This association is an effect of DBP concentration levels in different genetic variants. The Gc1F genotype has a low concentration of DBP with high affinity for vitamin D metabolites; consequently, low bioavailable vitamin D levels have been reported. ^16^ Therefore, we performed a cross-sectional study to determine the frequency distribution of DBP gene polymorphisms among ATB patients, LTBI patients, and individuals without TB infection in a Ugandan population.

## Methods

### Study design and study setting

This was a pilot study based on a previous cross-sectional study of 148 participants between the ages of 12-65 years of which 102 samples were conveniently selected. Details of this previous study have been reported elsewhere. ^17^ This study was nested from a larger study that was conducted in accordance with the Declaration of Helsinki, and approval was granted by the Makerere University School of Biomedical Sciences Higher Degrees Research Ethics Committee (SBS HDREC)/#SBS-637 on 25 ^th^ Jan 2019, Kiruddu Referral Hospital, and National Council of Science and Technology (HS2639) on the 31 ^st^ October 2019. All experimental protocols were approved by Makerere University SBS HDREC (#SBS-637) and the National Council of Science and Technology (HS2639), as guided by the Helsinki Declaration. Written informed consent was obtained from active TB patients at Kiruddu Hospital for study participation. Informed consent was obtained from the KTB household contacts, and the parents or guardians consented on behalf of the minors.

Following the inclusion and exclusion criteria, these samples were selected for genotyping of DBP gene polymorphisms. This was based on the availability of whole blood for ATB patients and peripheral blood mononuclear cells (PBMCs) for LTBI patients/individuals and those with no TB infection. After obtaining ethical approval and informed consent, Gen-expert-positive TB patients from Kiruddu Referral Hospital were enrolled, and samples of household contacts of LTBI Individuals with (QFN+ TST+) results and individuals with no TB infectionwho were (QFNTST-) from the Kampala TB (KTB) project were included in the study. Samples from patients with LTBI and those without TB infection were purposively selected. PBMC samples with adequate cellswere selected for genotyping, and samples with fewer cells were excluded. Based on this, 46 samples were excluded becauseof inadequate sample volume and the number of cells available for successful genotyping. Individuals withan HIV ^+^serostatus were not excluded from the study.

### DBP gene genotyping

The phenol-chloroform (PhCHCL _3_) method was used to extract DNA from whole blood samples of active TB patients, PBMCs fromLTBIpatients, and those with no TB infection.

Briefly 100 μl of 10% SDS were Dispensed in eppendorf tubes. 150μl of whole blood were then added and mixed by pipeting up and down. This was followed by incubation at 65°C for 10 min using a heat block. 100 μl of 3N Soduim Acetate were added and 5 vortexed vigorously. This was followed by addition of 700 μl of PhCHCL _3_. And 280 μl of PCR grade water. The tubes were inverted vigorously several times. they were then Centrifuged @ 13000 rpm for 30 min. 450 μl of the aqueous layer was Transferred to a new eppendorf tube. 1000 μl of absolute isopropanol (100%) was then added. DNA was precipitated at -80°C for 20min. this was followed by centrifuging at 14000 rpm for 30 minutes. The isopropanol was removed off leaving approx 50 μl. Add 700 μl of 70% isopropanol were added and Centrifuged @ 14000 rpm for 30 minutes. The 70% isopropanol was completely removed leaving the dry pellet. The DNA tubes were dried at 65°C. DNA was eluted in 100 μl of PCR H _2_O @ 65°C. It was then stored at – 80 °C for future use.

Agarose gel electrophoresis of human genomic DNA was performed using 1% agarose gel prepared by weighing and dissolving 1.5 g of agarose in 150 ml of 1x TAE (1% solution). The agarose was boiled thoroughly in a microwave oven for 3 minutes to allow thorough heating and mixing, and allowed to cool to 50°C at room temperature. 7.5 μl of 5 mg/ml ethidium bromide was added and mixed well by gentle agitation. The Agarose solution-ethidium bromide mixture was poured into an assembled gel casting tray with a comb attached and allowed to set at room temperature for approximately one hour. Upon setting, the gel was placed in to the electrophoretic tank and the combs vertically removed. 1x TAE buffer was poured in to the electrophoretic tank to just cover the gel. 5 μl of loading dye was added to 5 ul of each of the PCR product on the Para film, mixed and then loaded on to the wells in the gel.

While loading, the molecular weight marker was always loaded on the first lane and then the extracted human genomic DNA. The samples were run at 120 volts (constant voltages, variable current) for 30 mins. After 1 hr, the electrophoresis was stopped and the gel was carefully transferred to a UV trans illuminator for visualization.

**Figure 1.**
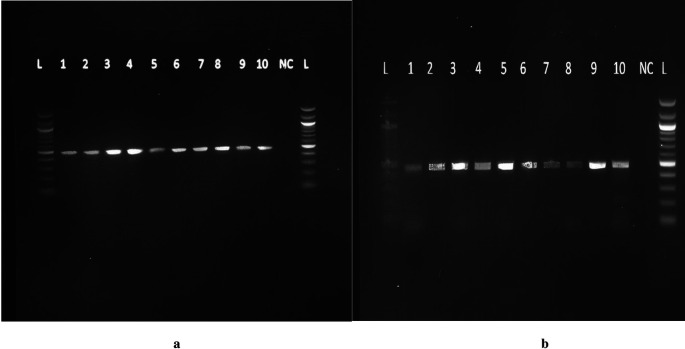
a: Agarose gel electrophoresis of the 483 bp DBP gene PCR product representative of active TB patients. Lanes: L=100bp DNA ladder, NC= Negative control, 1- 10=samples from active TB patients. b: Agarose gel electrophoresis of the 483bp DBP gene PCR product representative of LTBI and those with no TB infection. Lanes: L=100bp DNA ladder, NC= Negative control, 1-10=samples from LTBI and those with no TB infection. All the figures provided here are only found in a preprint and have not been published anywhere else. I therefore assume I do not need to request for copy right permissions.

Primers were purchased from Eurofins Genomics, Inc. Germany. The primer sequences were forward 5″AAATAATGAGCAAATGAAAGAAGAC3′ and reverse 5′ CAATAACAGCAAAGAAATGAGTAGA3′ with expected amplicons of approximately 483 bp. Mastermix preparation was performed from the pre-amplification room as follows: 25 μL of 2X Taq Master Mix, 2.5 μl of the reverse primer (6 pM) and 2.5 μl of forward primer (0.6 pM), and 15 μL of PCR water, making a volume of 45 μLforeach reaction. Forty-five microliters of the master mix and 5 μL of DNA were added to each of the PCR tubes. Five microliters of PCR water was added to the negative control tubeand transferred into the SimpliAmp Thermocycler for 40 cycles under the following programmed conditions: enzyme activation step 5 min at 95°C, denaturation for 20 s at 95°C, annealing for 45 s at 56°C, extension for 10 sat 72°C, Final Extension for 5 min at 72°C, and finally an infinite hold at 4°C. The amplicons were run on a 2% agarose gel, as previously described, and a product size of 483 bp was obtained ( Figure 1a and b). Under ambient conditions, the PCR products were sent for Sanger sequencing using the forward primer at ACGT in the United States of America. The ABI Big Dye Termination Kit (Applied Biosystems, USA) and the ABI prism 310 Genetic analyser (Applied Biosystems) was used. The sequenced chromatograms were obtained and cleaned up to remove low yield peaks. A BLAST query sequence was performed to confirm the DBP gene against that of the NCBI library. The gene products were named Homo sapiens Gc vitamin D binding protein (Gc), with sequence sizes between 414 bp and 448 bp with a percent identity of 98-99%. The DBP gene reference sequence (AH004448.2, Homo sapiens vitamin D-binding protein gene) was retrieved from the National Center for Biotechnology Information (NCBI). The raw DBP genesequences from our analysis were aligned to the reference genome. Variant filtering was performed in which low-read regions and errors were identified. Coverage, quality scores, and proximitywere also checked. Sites that differed from the reference genome and sequences were identified and sorted according to their nucleotide and amino acid composition. The detection of the presence of SNPs was performed by searching for the possible change in the codon GAT to GAG at position 416, representing the rs7041 variant, and ACG to AAG at position 420 for the rs4588 variant.

### Statistical analysis

The data were summarized using STATA software (Stata Corp. STATA 16.0, College Station, Texas, USA). Frequency and percentage (n [%])were used to determine the frequency distribution of the DBP gene. Pearson’schi-square test was used to compare the frequency of DBP among the study groups and a subanalysis of sex. Fisher’s exact test was used to test for significance among the groups. A potential deviation from Hardy–Weinberg equilibrium was performed using the dnaSP software. V5 ( http://www.ub.es/dnasp).

The p-value was considered significant at P < 0.05, with a 95% confidence interval.

## Results

### Socio-demographic description of study participants

Of these, 102 were genotyped, of which 52 were newly diagnosed ATB patients, 23 had LTBI and 27 had no TB infection. The median age of the study participants was 28years, and the majoritywere female (63 [61.2%]). A small proportion of these patients were HIV-positive, 9 (18.4%). Table 1 shows the social, demographic, and clinical characteristics of the study participants and more details of the study participants have been described elsewhere. ^17^

**Table 1.** Socio-demographic and clinical characteristics of study participants.

Participant characteristic	Frequency n( %)	Median (IQR)
Age (years)		28 (12,65)
18 and below	19(18.4)	
19-30	46(44.6)	
31-40	22(21.3)	
Above 40	15(14.5)	
Sex		
Female	63(61.2)	
Male	40(38.8)	
TB status		
No TB infection	27(26.4)	
Latent TB infection	23(22.5)	
Active TB	52(50.1)	
BCG scar		
No	47(46.1)	
Yes	55( 53.9)	
Alcohol consumption		
No	75(73.5)	
Yes	27 (26.5)	
Smoking		
No	96(94.1)	
Yes	6(5.9)	
HIV status		
Negative	83(81.4)	
Positive	19(18.6)	

n% represents the number of participants, IQR is interquartile range.

### Frequency distribution of the DBP rs7041 and rs4588 SNPs among active TB patients, LTBI, and those with no TB infection

**Figure 2.**
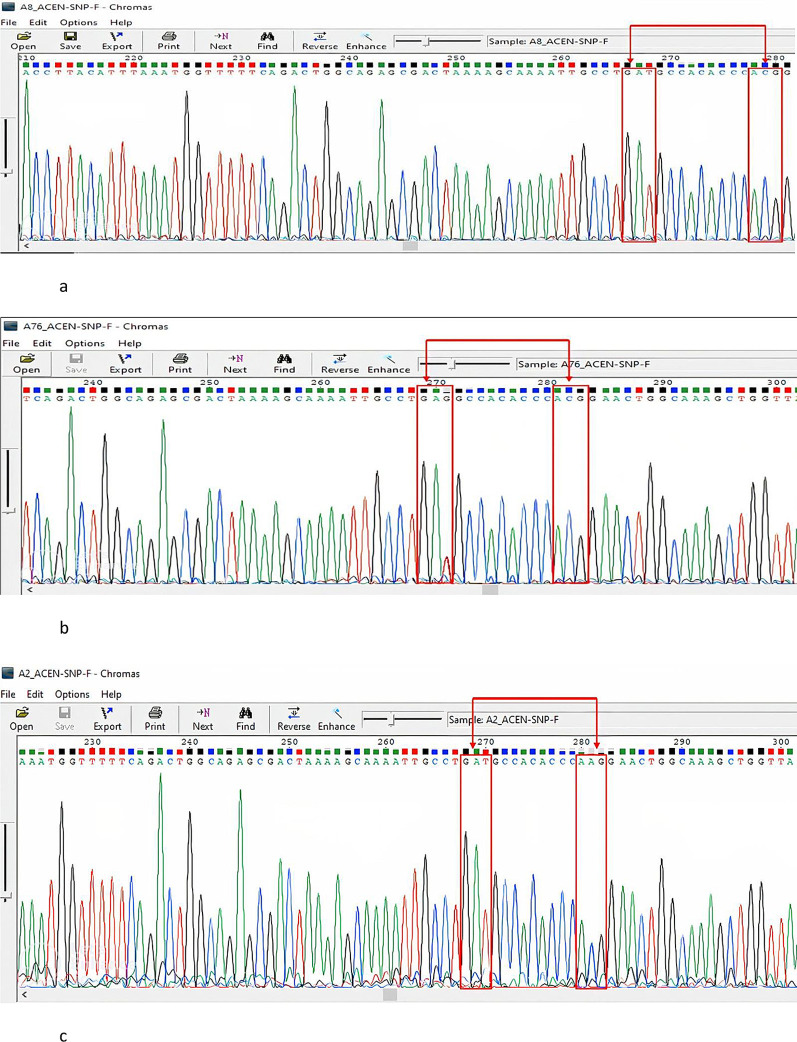
a: A Sanger sequencing representative chromatogram of the GAT and ACG (Gc1F) genotype. No conversion was observed in both SNPs rs7041and rs4588, The figure was generated using BioEdit 7.2 software http://www.mbio.ncsu.edu/BioEdit/bioedit.html by A.A. b: A Sanger sequencing representative chromatogram of the GAG and ACG (Gc1S) genotype. A conversion was observed in the rs7041 SNP GAT to GAG and no conversion noted in the rs4588 SNP. The figure was generated using. The figure was generated using BioEdit 7.2, http://www.mbio.ncsu.edu/BioEdit/bioedit.html by A.A. c: A Sanger sequencing representative chromatogram of the GAT and AAG (Gc2)genotype. No conversion was observed in the rs7041 SNP and conversion is noted in the rs4588 SNP from ACG to AAG. The figure was generated using BioEdit 7.2 http://www.mbio.ncsu.edu/BioEdit/bioedit.html by A.A.

A Gc1S reference sequence with the GAG codon at position 416 was retrieved from the NCBI database (Homo sapiens vitamin D-binding protein gene) for use. According to our search in BioEdit, all our sequences had the wild-type GAT codon at this position compared to the reference sequence. At position 420, all of our samples had an ACG codon, except for two samples that showed a conversion to AAG. Ninety-seven percent of the study population had rs7041 GAT and ACG for the rs4588 codons, 2% had GAT rs7041 and AAG rs4588, and 1% had rs7041 GAG and rs4588 ACG (Gc1S). Figure 2a- c show the details of this analysis and highlighted transformations. Therefore, the frequency distribution of the DBP genotypes in the study population was Gc1F, 97%; Gc, 2.2%; and Gc1S, 1%. The frequency distribution of the DBP genotypes among patients with TB was 96.2% Gc1f, 0% Gc1S, and 3.8% Gc2. Among the LTBI cases, 95.7% were Gc1F, 4.3% were Gc1S, and 0% were Gc2. For those without TB infection, the frequencies were Gc1F 100%, Gc1s 0% and 0% for Gc2. There was no statistically significant difference in the predominant Gc1F genotype among ATB patients, LTBI individuals, and those without TB infection (P=0.3). Notably, the participants with the Gc2 genotype were ATB patients with HIV coinfection. Furthermore, we alsofound that individuals with the Gc1S genotype hadLTBI. The genotype and allele distributions of the study participants are shown in Table 2 and Table 3, respectively. The Hardy-Weinberg equilibrium analysis was in equilibrium, D’=0, P=0.2

**Table 2.** Genotype and allele distribution of the DBP gene among ATB patients, LTBI, and those without TB infection.

GENOTYPES	ALLELES	Active TB patients	LTBI	Those with no TB infection	N Total/ P value
		N= 52	N=23	N=27	N=102
GCIF	GC1F rs7041(A)- rs4588(G)	50 (49%)	22 (21.6%)	27(26.4%)	P=0.6
GCIS	GC1S rs7041(C)- rs4588(G)	0 (0%)	1(1%)	0 (0%)	
GC2	GC2 rs7041(A)- rs4588(T))	2 (2%)	0(0%)	0 (0)%	

The frequency distribution of genotypes is represented as a percentage, where n is the number of participants and percentage. The P value was significant at < 0.05.

**Table 3.** Frequency distribution of DBP genotypes according to TB status.

DBP genotypes	TB patients n %=52	LTBI n %=23	Those with no TB infection n %=27	P value
Gc1F	50(96.2%)	22(95.7%)	27(100%)	0.29
Gc1S	0(0%)	1(4.3%)	0(0%)	
Gc2	2 (3.8%)	0(0%	0(0%)	

P value ≤ 0.05.

### Relatedness ofthe DBP reference gene sequence and study sequence data

**Figure 3.**
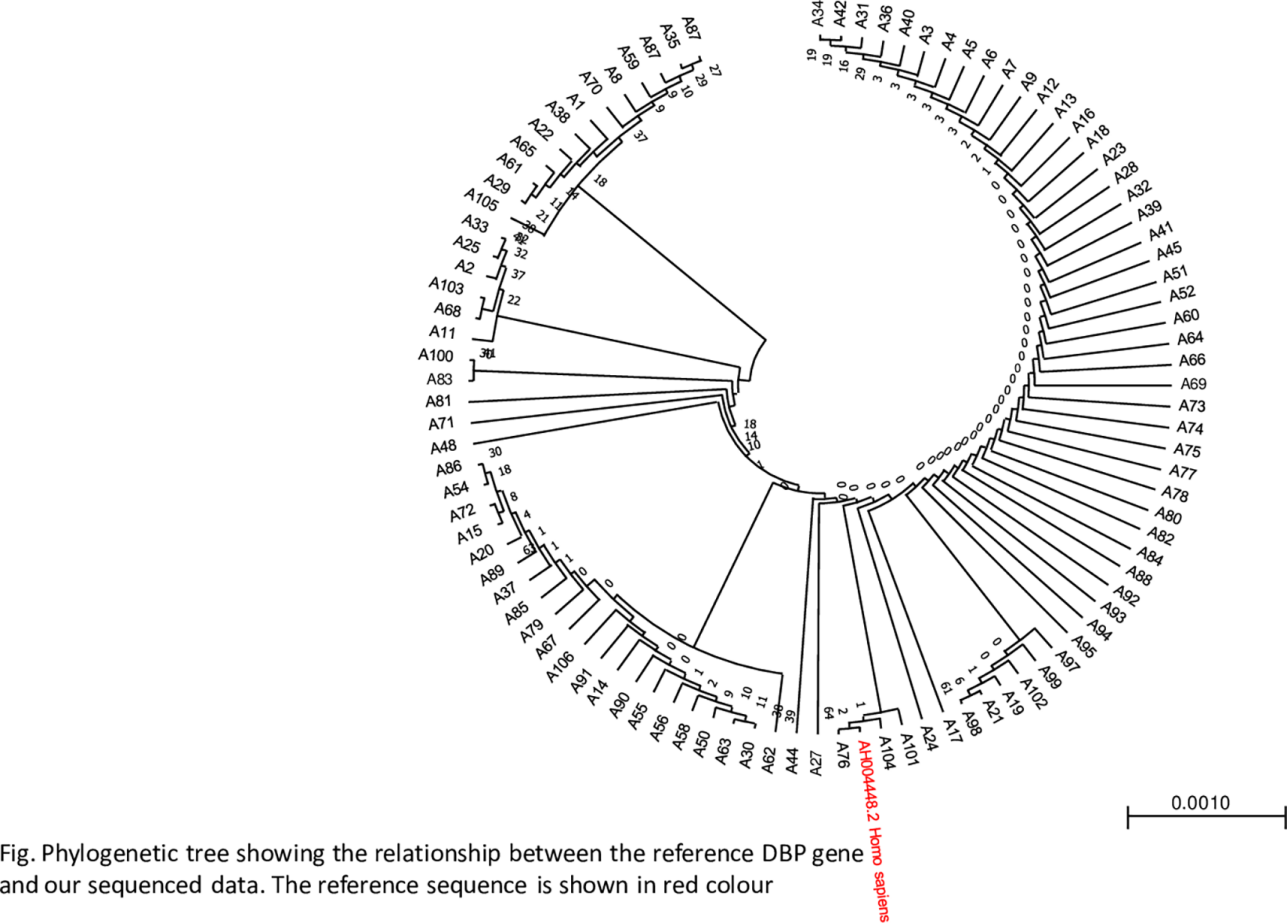
Showing a phylogenetic tree indicating the relationship between the reference gene and sequence data, The figure was generated using BioEdit 7.2 http://www.mbio.ncsu.edu/BioEdit/bioedit.html by A.A.

A phylogenetic tree was constructed to determine the closeness of the sequences using the maximum likelihood method. The phylogenetic tree revealed a close relationship between the samples and the reference genes, as shown in Figure 3.

## Discussion

DBP is highly polymorphic, with approximately 120 variants; however, the widely studied variants are the rs7041 and rs4588 SNPs from these three variant genotypes, Gc1F, Gc1S, andGc2. These genotypes arethe predominant source of diversity observed across different geographic locations and ethnicities. Well-documentedreports have focused on multiracial populations and lack adequate information regardinghomogeneous populations. Extensive research in population genetics has found that the frequency of the Gc1F genotype is predominantly found in Africans and African-Americans and that Gc2 is the lowest. ^18^ Our study showed a frequency distribution of 97% of the Gc1F genotype, 2% of the Gc2 genotype, and 1% of the Gc1S genotype in the population. This is consistent with the genotype frequency distribution ofAfrican Black populations. This finding is comparable to that of two West African studies in Gambia, with a nearly homogeneous population like ours. They reported a frequency distribution of 86.0% and 83.3%, respectively, and another study from South Africa reported 80.0%. ^11, 19, 20^ However, these studies had a larger sample size thanthe current study. Similarly, findings from a study among Black Americans and whites showed a frequency distribution of 92.7% for the Gc1F genotype among Blacks (2.1%) and Gc2 (2%). In contrast, the same study found a high-frequency distribution of Gc1S among the white population. ^18^ In contrast, a study performed among the Eurasian population found the Gc1F genotype to be the lowest (13.7%) and the highest was Gc1S. ^16^ Correspondingly, astudy from Finland reported a low frequency of Gc1F (3.7%). ^21^ The above observations show that DBP polymorphisms are ethnically based; therefore, diverse effects on vitamin D metabolites are likely to be observed. This study did not finda statistically significantdifference in the frequency distribution of the Gc1F DBP genotype among the three study groups (P=0.3). This finding is similar to that of a study from Pakistan that reported a non-significant association of DBP with TB (P=0.3). ^22^ However, we noted that the Gc2 genotype was only found among active TB patients with HIV coinfection. This finding is comparable to that of the previously mentioned South African study that reported an association between the Gc2 genotype and TB status among Asians. ^23^ Furthermore, a recent study in China exploring vitamin D pathwaygene polymorphisms foundthat the DBP Gc2 genotype was associated with progression to pulmonary TB. ^25^ Moreover, in our study, the Gc1S genotype was detectedin the LTBI group. Therefore, these findings suggest that the minor alleles in our population have a genetic association. The Gc1F genotype is predominant in the black population; therefore, it is worth mentioning that our population was consistent with the Hardy-Weinberg equilibrium, and the frequency distribution observed is possibly a representation of our study population. Consequently, in additiontogenetic predisposition, environmental, social, and economic factors may play a major role in TB susceptibility in the population.

Regarding the HIV sub-analysis, no statistical significance was found among the genotypes and TB status.

Considering the analysis of sex and the DBP gene, no statistical difference was observed in the frequency distribution among male and female participants (P=0.07). This observation is similar to thatof a study in India on TB patients. ^24^

We acknowledge that the small sample size and homogeneous population of the current study could have contributed to the less significant effect size needed to detect minor alleles, as observed. Therefore, future studies should considerlarger sample sizes to increase the probability of detecting minor alleles in the population. The strength of this study is that it is the first to determine DBP gene polymorphisms in the Ugandan population of active TB patients, LTBI, and household contacts, providing an adequate representation of TB status.

## Conclusion

The frequency distribution of the DBP and Gc1F genotypes was predominantly found in the study population, with no statistically significant difference among the ATB patients, LTBI patients, and those with no TB infection. However, the minor alleles, Gc2 andGc1S, may be associated with a higher risk of active TB and LTBI. Further research is warranted in a larger homogenous and heterogeneous population to adequately determine the functional significance ofminor alleles and their role in TB pathogenesis. This is important for TB control and prevention.

## Ethical approval and informed consent

This study was nested from a larger study that was conducted in accordance with the Declaration of Helsinki, and approval was granted bythe Makerere University School of Biomedical Sciences HigherDegrees Research Ethics Committee (SBS HDREC)/#SBS-637 on 25 ^th^ Jan 2019, Kiruddu Referral Hospital, and National Council of Science and Technology (HS2639) on the 31 ^st^ October 2019. All experimental protocols were approved by Makerere University SBS HDREC (#SBS-637) and the National Council of Science and Technology (HS2639), as guided by the Helsinki Declaration. Written informed consent was obtained from active TB patients atKiruddu Hospital for study participation. Informed consent was obtained from the KTB household contacts, and the parents or guardians consented on behalf of the minors.

## Authors’ contributions

Conceptualization: **E.L.A.,** Data curation: **E. L. A.,** Formal analysis: **E. L. A., A. A., R.B. S. N., A. O.,** Funding Acquisition: **E.L.A., D.P. K.,** Investigation: **E.L.A.,** Project Administration: **E.L.A., I.A.B., W.W., D.P.K., M.L.J.,** Methodology: **E. L. A., A. A., R.B.S.N., A.O. K. B.,** Resources: **E.L.A., I, A.B., A.A., R.B. S. N.,** Software: **M. B., A.A. K. B.** Supervision: **W. W., D.P. K., I. A. B., M L. J.,** Validation: **E.L.A.,** **W. W., D.P. K., I. A. B., M L. J.,** Visualization: **E.L.A., A.A.R.B. S. N., M.B., K.B., A.O.,** Writing – original draft: **E. L. A.,** Writing – review & editing: All authors reviewed the manuscript.

## Data Availability

The dataset that was generated and analyzed during the current study is available in GenBank with accession numbers BankIt2605152: *OP032652 - OP032748* https://www.ncbi.nlm.nih.gov/nuccore/OP032652.1/. The project contains Figshare: Ester supplimentary data.zip. https://doi.org/10.6084/m9.figshare.28234484.v3. ^25^ Data are available under the terms of the Creative Commons (CC0 1.0 Public domain dedication), 4 Copyright: © 2025 Ester Acen et al.
