# Supplementary material for: Vitamin D binding protein rs7041 and rs4588 gene polymorphisms in Ugandan tuberculosis patients and household contacts: A pilot study
Source: F1000Res. 2025 May 21;14:154. Originally published 2025 Feb 4. [Version 2] doi: 10.12688/f1000research.160839.2 (PMC12699214; doi:10.12688/f1000research.160839.2)
Supplement: Peer review history

# Reviewer report: Reviewer response for version 2

**DOI:** 10.5256/f1000research.182062.r434456

**Published:** 2025-12-11

**Authors:** Habtamu Milkias Wolde

**Affiliations:** 1 School of Medicine, Department of Precision Medicine, SungKyunKwan University, Suwon, Seoul, South Korea

**Comment**

 This is an interesting study that investigates the distribution of Vitamin D binding protein gene polymorphism among patients with active TB, latent TB and individuals with no TB infection in Uganda. It’s known fact that Vitamin D plays an important role in the immune systems response to TB and DBP gene polymorphisms can affect Vitamin D levels in the body and potentially increasing individuals’ susceptibility to TB. And the authors found no significant difference in the level of polymorphisms among the study groups which is again an interesting null finding. However, there are certain issues that need to be clarified before the manuscript is ready to be indexed, which I describe below as questions or suggestions.

  

1. Introduction

- On the first line of the introduction section, the phrase “between 200-350 per 100 cases…” seems to be incorrect. Did you mean per 100,000 cases or some other denominator?
- On the 6th line the following phrase “…reported among adult TB patient” patient should be made plural by adding “s”.
- There are similar editorial issues the authors should carefully look into

 

1. Methods

- Regarding the sample size, the study has included only 102 participants and out of these 46 samples were excluded. Would this size be sufficient enough reach to the conclusion?
- The samples were selected based on a convenience sampling method which is a non-random method? So, do you think this is going to affect generalizability of the study to the Ugandan population?
- Fisher’s exact test is preferred for small samples sizes but power calculations should have been done. With small sample, the study might be underpowered to detect modest risk/effect of the polymorphism on TB disease.
- Though the authors mostly conducted descriptive analyses of the data. But associations between variables/cause and effect are better assessed though stronger methods including regression models. Why did you restrict the analyses to descriptive stats only?
- Measuring the level of Vitamin D in the participants would have aided the analysis acting as a direct measure (e.g., comparison of blood plasma level) in addition to the genetic analysis.

 

1. Results and discussion

- It would be insightful to discuss about the effect of the polymorphism on the different groups of participants including whether it accelerates progression of latent TB into active TB or whether it predisposes healthy individuals to be susceptible to TB infection.

**Minor Issues**

- Grammar and clarity, many sentences require editing for clarity.
- Methods section is too long. It should be shortened unless critical.
- Acronyms should be defined once and then used consistently (e.g., ATB, LTBI)
- Some typographical issues like missing spaces, repeated words, uppercase errors.

Is the work clearly and accurately presented and does it cite the current literature?

Yes

If applicable, is the statistical analysis and its interpretation appropriate?

Partly

Are all the source data underlying the results available to ensure full reproducibility?

Yes

Is the study design appropriate and is the work technically sound?

Yes

Are the conclusions drawn adequately supported by the results?

Partly

Are sufficient details of methods and analysis provided to allow replication by others?

Yes

Reviewer Expertise:

My broader area of expertise is Tropical and Infectious Diseases but I specialize in diseases such as Tuberculosis and Measles.

I confirm that I have read this submission and believe that I have an appropriate level of expertise to confirm that it is of an acceptable scientific standard.

# Reviewer report: Reviewer response for version 2

**DOI:** 10.5256/f1000research.182062.r386374

**Published:** 2025-06-24

**Authors:** Dina Keumala Sari

**Affiliations:** 1 Department of Nutrition, Faculty of Medicine, Universitas Sumatera Utara, Medan, Indonesia

I think this paper already fulfill the criterias and corrections.

 I have no further comments.

Is the work clearly and accurately presented and does it cite the current literature?

Yes

If applicable, is the statistical analysis and its interpretation appropriate?

Yes

Are all the source data underlying the results available to ensure full reproducibility?

Yes

Is the study design appropriate and is the work technically sound?

Yes

Are the conclusions drawn adequately supported by the results?

Yes

Are sufficient details of methods and analysis provided to allow replication by others?

Partly

Reviewer Expertise:

Vitamin D and polimorphism of Vitamin D receptors.

I confirm that I have read this submission and believe that I have an appropriate level of expertise to confirm that it is of an acceptable scientific standard.

# Reviewer report: Reviewer response for version 1

**DOI:** 10.5256/f1000research.176796.r369604

**Published:** 2025-03-13

**Authors:** Ramalingam Bethunaickan

**Affiliations:** 1 ICMR-National Institute for Research in Tuberculosis, Chennai, India

The authors studied Vitamin D binding protein gene polymorphisms in Ugandan tuberculosis patients and household contacts and reported no significant differences in DBP genetic polymorphisms among the study groups. This study needs to address the following points for better clarity.

**Major comments:**

 1. This study was conducted in small sample size. The 102 samples were selected from previous cross-sectional study among which approximately 50% of the samples were excluded and stated that due to inadequate sample volume. For genotyping maximum 2ml blood is adequate for DNA extraction to carry out the experiment, hence those excluded can be done with lesser blood volume using cost effective methods like PCR-RFLP. Moreover, in this study individuals with an HIV+ serostatus were not excluded, then how authors would eliminate those confounders from the study results?

 2. This study lack to study different genetic models and better fit model for their study population. Haplotype analysis needs to be done.

 3. VDBP gene variants shown to be associated with serum vitamin D levels. Hence, for better understanding vitamin D and VDBP levels needs to be correlated with gene variants to known its association with the disease.

 4. The authors stated that minor alleles appear to be associated with an increased risk of active and latent TB. However, no such significant association was noted in analysis and the frequencies in TB were 0% Gc1S, and 3.8% Gc2. Among the LTBI cases, 4.3% were Gc1S, and 0% were Gc2. For those without TB infection, the frequencies were Gc1s 0% and 0% for Gc2.

 This needs to be confirmed with larger sample size.

**Minor comments:**

 1. Include Gender based association Table.

 2. Gene name mention in italics

 3. In methods section, give sub-titles for DNA isolation, Gel electrophoresis, and Sanger methods.

 4. Conclusion, minor allele association needs to be removed.

 5. Grammar and typological errors needs to be checked throughout the manuscript.

Is the work clearly and accurately presented and does it cite the current literature?

Yes

If applicable, is the statistical analysis and its interpretation appropriate?

Yes

Are all the source data underlying the results available to ensure full reproducibility?

Yes

Is the study design appropriate and is the work technically sound?

Yes

Are the conclusions drawn adequately supported by the results?

Yes

Are sufficient details of methods and analysis provided to allow replication by others?

Yes

Reviewer Expertise:

Immunology- Immunogenetics- Nutrition- SLE and Tuberculosis

I confirm that I have read this submission and believe that I have an appropriate level of expertise to confirm that it is of an acceptable scientific standard, however I have significant reservations, as outlined above.

# Reviewer report: Reviewer response for version 1

**DOI:** 10.5256/f1000research.176796.r369603

**Published:** 2025-03-11

**Authors:** Dina Keumala Sari

**Affiliations:** 1 Department of Nutrition, Faculty of Medicine, Universitas Sumatera Utara, Medan, Indonesia

**Title**: please add specific with the reference SNPs that stated in this study to clear which gene that involved in this research.

**Abstract:**

 In the introduction: please state about Gc1F genotype and its Reference SNPs.

 In the methods: please clear when and where this research conducted.

 In conclusions: no need to state about recommended larger studies, because this study should be collect the data by counting the right sample size.

 Keywords: please choose other keywords that are not in the title to increased the search engine to the reader.

**Main text:**

 Introduction: state about prevalence of Tuberculosis and vitamin D deficiency in Uganda, is it still main infectious problem? show the important or urgent of this study. So, it became the based of the research conducted.

 Add more references that support the lower vitamin D level in this population. Is there any information about sun exposure or vitamin D intake in this area?

**Methods:**

 Please add about the inclusion and exclusion criteria for each groups, the information about inclusion and exclusion based on previous study only explain about household contact subject based on *BMC Infectious Diseases* 2015 **15**, Article number: 438  Biraro et al. study and *PLoS One.* 2022 Acen et al [2022 (Ref-1)]: https://doi.org/10.1371/journal.pone.0272788. Not clear enough.

 Is there any  data about vitamin D level serum in this research? Why this research did not assess vitamin D serum level whether 25(OH)D serum level or 1.25(OH)D serum level as the most active metabolite.

**Statistical analysis:**

 Why choosing Fisher's exact test than Chi-Square test, is there any adjustment for this decisions?

**Results:**

 Please revise the P symbol into lettercase and itallic

 Is there any difference between ATB-LTBI patient and group with no TB infection? 

**Discussion:**

 Please add more discussion about related of this SNPs related to the Vitamin D level. 

 Please stated also about sun exposure in Uganda and also is there any possibility all the subject avoid the sun exposure.

 There are a statement about HIV, is this related to the lower vitamin D level? 

 Is there any co founding factor in this research? how to control it?

 Is there superiority of these two genotypes? is it okay if we only check for one genotype?

 state about TB control and prevention.

**Conclusions:**

 Please move the last statement of conclusions to discussion and add more information.

Is the work clearly and accurately presented and does it cite the current literature?

Yes

If applicable, is the statistical analysis and its interpretation appropriate?

Yes

Are all the source data underlying the results available to ensure full reproducibility?

Yes

Is the study design appropriate and is the work technically sound?

Yes

Are the conclusions drawn adequately supported by the results?

Yes

Are sufficient details of methods and analysis provided to allow replication by others?

Partly

Reviewer Expertise:

Vitamin D and polimorphism of Vitamin D receptors.

I confirm that I have read this submission and believe that I have an appropriate level of expertise to confirm that it is of an acceptable scientific standard, however I have significant reservations, as outlined above.

## References

[1] : Evaluation of circulating serum cathelicidin levels as a potential biomarker to discriminate between active and latent tuberculosis in Uganda. *PLoS One* .2022;17(8) : 10.1371/journal.pone.0272788 e0272788 10.1371/journal.pone.0272788 36018845 PMC9416991
